# Association between pro-inflammatory diet and liver cancer risk: a systematic review and meta-analysis

**DOI:** 10.1017/S1368980023002574

**Published:** 2023-12

**Authors:** Kaixia Chen, Fen Yang, Xinhong Zhu, Guiyuan Qiao, Chunmei Zhang, Junxiu Tao, Xiaolian Gao, Mingzhong Xiao

**Affiliations:** 1 School of Nursing, Hubei University of Chinese Medicine, Wuhan 430065, China; 2 Institute of Liver Diseases, Hubei Key Laboratory of the theory and application research of liver and kidney in traditional Chinese medicine, Hubei Provincial Hospital of Traditional Chinese Medicine, Wuhan 430061, China; 3 Affiliated Hospital of Hubei University of Chinese Medicine, Wuhan 430074, China; 4 Hubei Province Academy of Traditional Chinese Medicine, Wuhan 430074, China

**Keywords:** Liver cancer, Pro-inflammatory diet, Dietary inflammatory index, Mate-analysis

## Abstract

**Objective:**

This systematic review aimed to investigate the association between dietary inflammatory potential and liver cancer to provide evidence regarding scientific dietary health education.

**Design:**

Systematic review and meta-analysis.

**Setting:**

A comprehensive literature review was conducted to identify case–control or cohort studies that involved dietary inflammation index (DII)/empirical dietary inflammation pattern (EDIP) and liver cancer in PubMed, EMBASE, Cochrane, and Web of Science databases. Using a combination of DII/EDIP and liver cancer as the search terms, the associations between DII/EDIP and liver cancer were then assessed.

**Participants:**

Three case–control studies and two cohort studies were brought into the meta-analysis, with 225 713 enrolled participants.

**Results:**

Meta-analysis of categorical variables showed that DII/EDIP in the highest category increased the risk of liver cancer compared to DII/EDIP in the lowest category (relative risk (RR) = 2·35; 95 % CI 1·77, 3·13; *P* = 0·000) and with low heterogeneity across studies (I^2^ = 40·8 %, *P* = 0·119). Meta-analysis of continuous variables showed that significant positive association between liver cancer and DII/EDIP scores (RR = 1·24; 95 % CI 1·09, 1·40; *P* = 0·001), and no heterogeneity (I² = 0·0 %, *P* = 0·471). Stratified according to the study design, there was a significant positive association between liver cancer and DII/EDIP scores in both cohort studies (RR = 2·16; 95 % CI 1·51, 3·07; *P* = 0·000) and case–control studies (RR = 2·75; 95 % CI 1·71, 4·41; *P* = 0·000).

**Conclusion:**

The higher the DII/EDIP score, the higher the risk of liver cancer. This finding may have prominent implications for the general population.

According to the report in 2020, the number of new cases of liver cancer in the world was approximately 905 700, ranking sixth among new malignant tumours, and the number of deaths was approximately 830 200, ranking third among malignant tumours^(1)^. With a similar trend, the disease burden of liver cancer in China is particularly serious. It was reported that the number of new cases of liver cancer in China was about 41 100, ranking fifth among new malignant tumours, and the number of death cases was about 391 200, ranking second among malignant tumours^(2)^. The WHO predicts that one million people will die of liver cancer each year by 2030^(3)^. Hence, the seriousness and risk of liver cancer have received great attention^(4,5)^.

Liver cancer risk factors include family history, obesity, type 2 diabetes, metabolic syndrome, viral hepatitis, and other chronic liver diseases, and all of these risk factors have an inflammatory profile^(6–8)^. Chronic inflammation is closely related to the development of cancer. A large number of epidemiological investigations have suggested that chronic inflammation plays an important role in the tumorigenesis of liver cancer, primarily initiating and promoting its progression, with approximately 80 % of cases progressing from chronic hepatitis to fibrosis and ultimately to malignancy^(9)^. Chronic inflammation drives immune cells to activate inflammatory cytokines^(10,11)^, which promotes the survival, proliferation, growth, and invasion of precancerous cells, and maintains tumour-related inflammation by inducing the production and attraction of new chemokines, ultimately leading to to tumour progression and spread^(12,13)^. Despite advances in the treatment of liver cancer, it remains one of the most common causes of cancer-related death worldwide^(14)^. Therefore, the prevention of liver cancer is particularly important.

Changing diets to prevent weight gain or promote weight loss could potentially reduce the risk of liver cancer/disease. Prior studies^(15,16)^ have indicated that the inflammatory pathway of the human body can be affected by certain specific nutrients and dietary patterns with anti-inflammatory or pro-inflammatory properties. To present the diet inflammatory potential more intuitively, some tools to describe the diet inflammatory potential have been invented in recent years, such as dietary inflammation index (DII) and empirical dietary inflammation pattern (EDIP). The DII was calculated based on dietary intake data^(17)^. Z-scores and central percentiles for each food parameter in each individual in the study or survey are calculated based on the world mean and standard deviation for the forty-five food parameters after obtaining dietary intake data for each individual in the study or survey. Each food parameter and its corresponding ‘overall food parameter-specific inflammatory effect score’ were multiplied by the central percentile of each food parameter to obtain the ‘food parameter-specific DII score’. Finally, all the ‘food-specific parameter DII scores’ were summed to obtain the individual ‘overall DII score’. The EDIP^(18)^ was derived based on thirty-nine predefined food groups from FFQ using reduced-rank regression followed by stepwise linear regression models. DII and EDIP are designed to assess the overall inflammatory potential of the whole diet, and both have higher scores indicating higher dietary inflammation. In this regard, the DII was derived from the literature and used on a population basis to compare the inflammatory potential of diets in different populations, an ‘*a priori*’ pattern score, which can be used to validate six inflammatory markers (i.e. IL-1*β*, IL-4, IL-6, IL-10, TNF-*α* and C-reactive protein)^(17)^. The EDIP is a hypothesis-driven, empirically derived dietary pattern that assesses diet quality based on its inflammatory potential, is an ‘*a posteriori*’ pattern score, and best predicts three inflammatory biomarkers (i.e. IL-6, C-reactive protein and TNF-*α* receptor-2)^(18)^. According to the literature, EDIP is a simplified version of the DII, both are indicators of dietary inflammation, reflecting the overall dietary inflammatory potential, so DII and EDIP were combined for the analysis in this study. Based on these assessment tools, previous studies have found that dietary patterns with a pro-inflammatory diet are highly associated with the risk of many diseases, such as diabetes^(19,20)^, breast carcinoma^(21)^, ovarian cancer^(22)^, obesity^(23)^, CVD^(24)^, mild cognitive impairment and dementia^(25)^. Likewise, some studies have investigated the role of dietary inflammation in the development of liver cancer; however, the conclusions are inconsistent. In this case, we conducted a meta-analysis of observational studies to evaluate the association between a pro-inflammatory diet and liver cancer risk.

## Methods

### Search strategy

The literature search was conducted using the PICOS (Population, Intervention, Comparison, Outcome and Study design) criteria. Participants (P): adults aged ≥ 18 years and free of liver cancer at baseline for cohort studies or receiving treatment for cancer; a validated dietary questionnaire was done for the study participants. Interventions (I): dietary score (DII or EDIP). Comparisons (C): highest *v*. lowest level (or the reverse) or per one-unit increase or decrease of the dietary score (DII/EDIP). Outcomes (O): risk of developing liver cancer. Study design (S): prospective cohort studies and case–control studies. In this work, we searched electronic databases Cochrane, PubMed (US National Library of Medicine, National Institute of Health), EMBASE and Web of Science as of 1 April 2022. Medical Subject Headings (MESH) combined with free search terms related to the dietary inflammatory index of correlation (dietary inflammatory index, inflammatory diet, pro-inflammatory diet, anti-inflammatory diet, DII, dietary inflammatory pattern, inflammatory potential of diet and dietary score) and liver cancer (liver neoplasm, hepatic neoplasm, liver neoplasm*, cancer of liver, hepatocellular cancer*, hepatic cancer*, liver cancer, cancer of the liver, hepatic, neoplasm*, liver, cancer*, hepatocellular, carcinoma* and tumour*). The meta-analysis was restricted to articles published in English only with no restrictions on country of origin. We checked all titles, abstracts, and keywords and read the full text if necessary.

### Study selection

Studies were considered eligible if: (1) they were observational studies (cohort or case–control), (2) they assess the association of DII/EDIP with liver cancer onset or death, not included according to food supplements, (3) they provide 95 % OR, hazard rate (HR) or relative risk (RR), (4) the studies conducted through humans, and (5) the risk estimates with the highest DII/EDIP score compared to the lowest risk estimate were reported for data or continuity data. If the data were repeated, there may be multiple articles using the same data source, and the articles with the most complete data were included. Studies were excluded if (1) repeat reports, (2) reviews or expert comments, etc., or (3) incomplete data, if no full text is available.

### Data extraction and quality assessment

Two reviewers independently reviewed titles, abstracts, and full text of all studies retrieved from electronic databases and provided specific search strategies for relevant articles. If there is a difference between the two reviewers, the third party will resolve it and reach a consensus. For each study, we extracted the following population characteristics and study data: the first author’s surname, year of publication, country, study design, baseline sample size, sex, age range/mean (year), dietary score/index evaluation, adjustment for the covariate, risk estimate with most fully adjusted, adjustment for the covariate, follow-up (years) and Newcastle–Ottawa scale (NOS) scores. To assess the methodological quality of eligible studies, we used the NOS^(26)^, in which quality was judged according to selection (four stars), comparability (two stars) and outcome (three stars). Studies of seven stars or more indicated high quality, and all articles included in this meta-analysis were of high quality with a score of 7 or above.

### Statistical analysis

The original OR and HR reported in the article were considered equivalent estimates of RR. We calculated pooled RR and 95 % CI for DII/EDIP the highest *v*. the lowest categories in categorical variables. Meanwhile, the pooled RR and 95 % CI were calculated for continuous DII/EDIP data in continuous variables. All data in this study were analysed by Stata 17.0 software. The Q-statistic was used to evaluate the presence of between-studies heterogeneity, and I² statistics were used to calculate the size of the variation between studies due to heterogeneity^(27)^. Meanwhile, I² > 50 % indicating high heterogeneity. The random-effects model was selected due to significant heterogeneity; if not, the fixed-effect model was performed to combine results. This study selected the fixed-effect model for analysis due to low heterogeneity (I² < 50 %). We conducted a study to extract multiple data (including age, sex and stratification by different cohorts). Subgroup analyses were based on study design, and one study was excluded in each turn by a sensitivity analysis to investigate the robustness of summary results, and we combined the remaining DII studies.

## Results

### Literature search and basic characteristics of studies

**Fig. 1:**
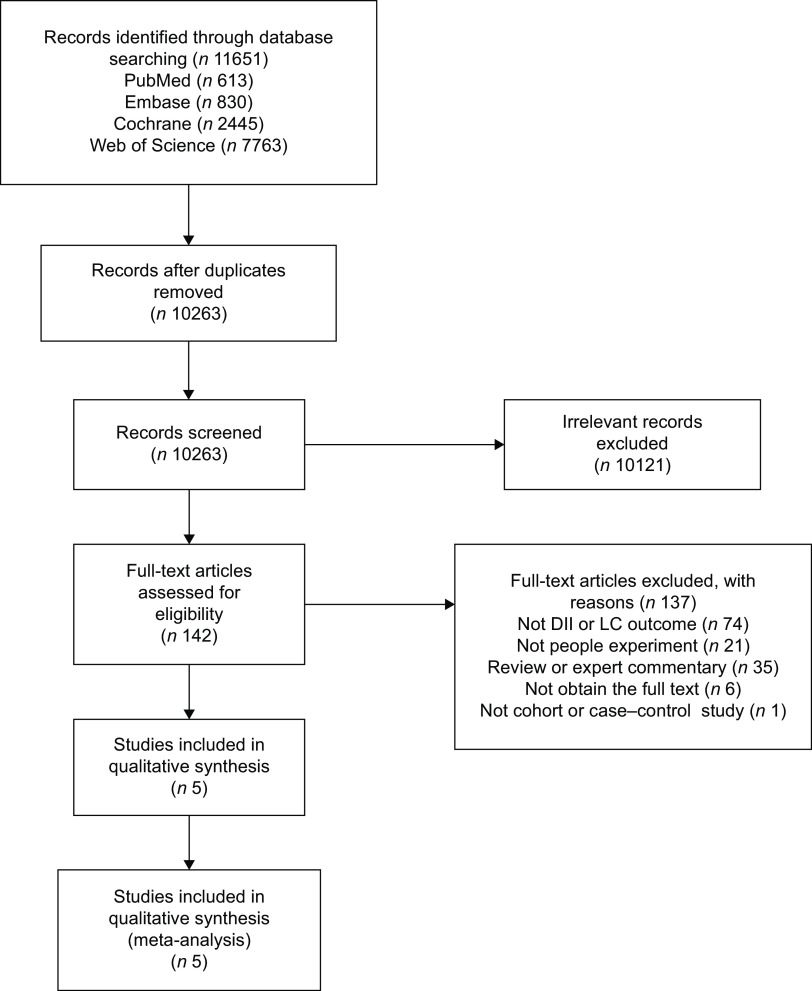
Flow chart of literature screening.

A total of 11 651 articles were retrieved from the database, and 10 263 remained after eliminating duplicates. Among them, 10 121 irrelevant articles were excluded by their titles and abstracts, and 142 related articles were reviewed in full. Excluding those irrelevant qualitative studies (*n* 137), five quantitative articles were left and thus evaluated for this meta-analysis. The detailed retrieval and screening process of the search is shown in Fig. 1. The five articles included studies^(28–32)^ that were published between 2016 and 2021, covering 225 713 individuals in total. Among all five studies, four of them^(29–32)^ assessed the potential of dietary inflammation using the DII score, and one study^(28)^ was evaluated using the EDIP score. In the three studies^(29,31,32)^, DII scores were evaluated by a FFQ, moreover, one EDIP^(28)^ was also assessed using the FFQ, and one^(30)^ was assessed by Diet History Questionnaire (DHQ). Two studies^(28,30)^ came from USA, two^(29,32)^ in Italy and one^(31)^ in China. Three studies^(29,31,32)^ were case–control design, and two^(28,30)^ were cohort studies. All five studies included achieved assessment with a 7/8-star score of NOS, and the quality evaluation of the pieces of literature was high. More detailed information on the five included studies is provided in Table 1.

**Table 1:** Basic characteristics of included studies

Studies	Country	Study design	Baseline sample size	Sex, age range/mean (year)	Dietary score/index evaluation	HR or OR (95 % CI)	Adjustment for covariate	Follow-up (years)	NOS scores
Yang W et al. (2021)	America	Cohort	119 316	Female, 30–55 Male, 32–87	EDIP: 39-item FFQ	HR tertile 3 *v.* 1: 2·03(1·31, 3·16)	Age, race, sex, BMI, physical activity, MET-h/week, type 2 diabetes mellitus, regular aspirin use, past smoking, current smoking, alcohol, total coffee intake and total energy intake	25·6	8
Accardi G et al. (2019)	Italy	Case–control	Case (liver):184 Con (liver): 404	Case (184): female, more than 50; male, more than 50 Con (404): female, 35–70; male, 35–70	DII: 78-item FFQ	OR (overall): 1·28 (0·97, 1·67)	Tobacco smoking, maximal lifetime alcohol intake, BMI, chronic infection with hepatitis B or C virus, and diabetes	NA	7
Zhong G C et al. (2020)	America	Cohort	103 902	Female, 55–74 Male, 55–74	DII: 137-item DHQ	HR tertile 3 *v.* 1: 2·57 (1·44, 4·60)	Age (years), sex, BMI, energy intake from diet, trial arm, ethnic group, education, smoking, alcohol drinking, diabetes, history of hepatitis or cirrhosis, and family history of liver cancer	13 or up to 31 December 2009	8
Wang X Y et al. (2018)	China	Case–control	Case: 659 Con: 659	Female, 18–80 Male, 18–80	DII: 79-item FFQ	OR (continuous): 1·19 (1·00, 1·42) OR tertile 3 *v.* 1: 3·22 (1·30, 7·98)	Sex, age, energy intake, BMI, physical activity, marital status, smoking status, alcohol drinking, education level and household monthly income per capita, and HBV infection status	NA	8
Shivappa N et al. (2016)	Italy	Case–control	Case:185 Con:404	Case (185): female, 43–84; male, 43–84 Con (404): female, 40–82; male, 40–82	DII: 63-item FFQ	OR (continuous): 1·24 (1·02, 1·51) OR tertile 3 *v.* 1: 2·43 (1·27, 4·68)	Sex, age, BMI, smoking, alcohol consumption, education (year) and hepatitis infection	NA	7

HR, hazard ratio; NOS, Newcastle–Ottawa scale; EDIP, empirical dietary inflammation pattern; DII, dietary inflammatory index; NA, not available; DHQ, Diet History Questionnaire; HBV, Hepatitis B Virus.

### Meta-analysis of the dietary inflammatory potential index and liver cancer risk

**Fig. 2:**
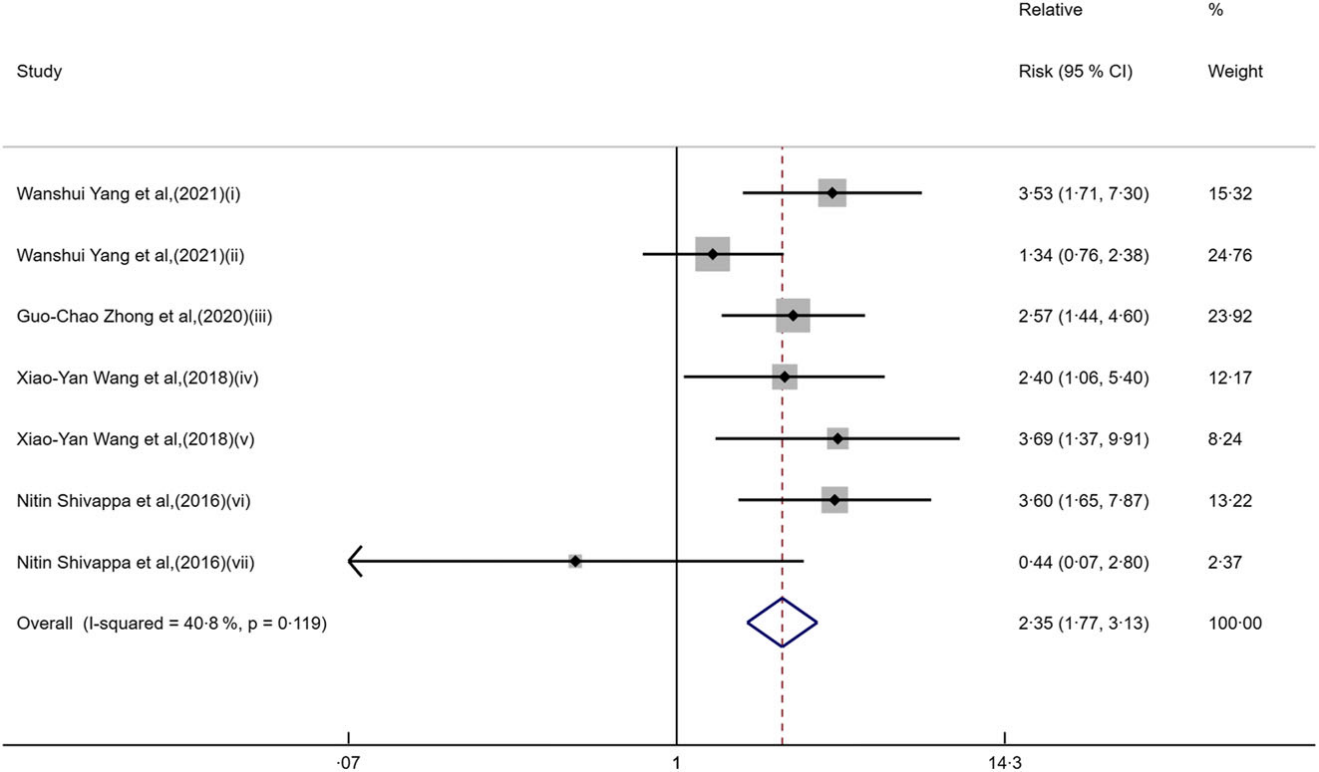
Forest plot for the primary outcome of the category data studies.

**Fig. 3:**
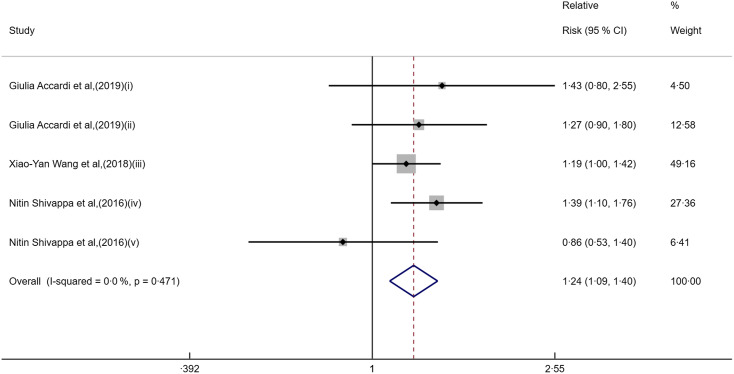
Forest plot for the primary outcome of the continuous data studies.

This meta-analysis included five pieces of literature, with a total of twelve data points, including seven categorical and five continuous data points, which were analysed separately, described as follows: in categorical study data (Fig. 2), Yang W et al. conducted studies (i) on the nurses’ health study (NHS) cohort population and (ii) on the health professionals follow-up study (HPFS) cohort population. Zhong G-C et al. conducted studies (iii) for people aged 55–74 years. Wang X Y et al. conducted studies (iv) on individuals aged under 60 years and (v) on individuals aged 60 years and older. Shivappa N et al. conducted studies (vi) on male data and (vii) on female data. In continuous study data (Fig. 3), Accardi G et al. carried out studies (i) for people aged 50–64 years and (ii) for those aged 65 years and older. Wang X Y et al. conducted studies (iii) for people aged 18–80 years. Shivappa N et al. conducted studies (iv) on male data and (v) on female data. When the pooled RR of the seven categorical data (Fig. 2 and Table 2) was used to assess the association between DII/EDIP and liver cancer, the results showed that the highest category increased the risk of liver cancer compared with the lowest category (RR = 2·35; 95 % CI 1·77, 3·13; *P* = 0·000). Meanwhile, there was low heterogeneity among studies in the categorical data (I² = 40·8 %, *P* = 0·119). The study design may partly explain the underlying heterogeneity in these studies. When the pooled RR of the five continuous data (Fig. 3 and Table 2) was used to assess the association between DII/EDIP and liver cancer, the results showed a significant positive association between liver cancer and inflammatory index in the diet (RR = 1·24; 95 % CI 1·09, 1·40; *P* = 0·001). The inter-study heterogeneity of continuous data was zero (I² = 0·0 %, *P* = 0·471), which may be due to the small sample size, resulting in insufficient statistical power to detect significance.

**Table 2:** Meta-analysis of the dietary inflammatory potential index and liver cancer risk, subgroup analyses stratified by study design

					Heterogeneity			
Group and subgroup	No. of studies	RR	95 % CI		*I* ^*2*^ (%)	*P*	*Z*-value	*P*
Overall (category)	7	2·35	1·77	3·13	40·8	0·119	5·90	0·000
Overall (continuous)	5	1·24	1·09	1·40	0·0	0·471	3·38	0·001
Subgroup analysis (category)								
Study design								
Cohort	3	2·16	1·51	3·07	58·3	0·091	4·24	0·000
Case–control	4	2·75	1·71	4·41	36·1	0·195	4·19	0·000

RR, relative risk.

### Sensitivity analyses

**Fig. 4:**
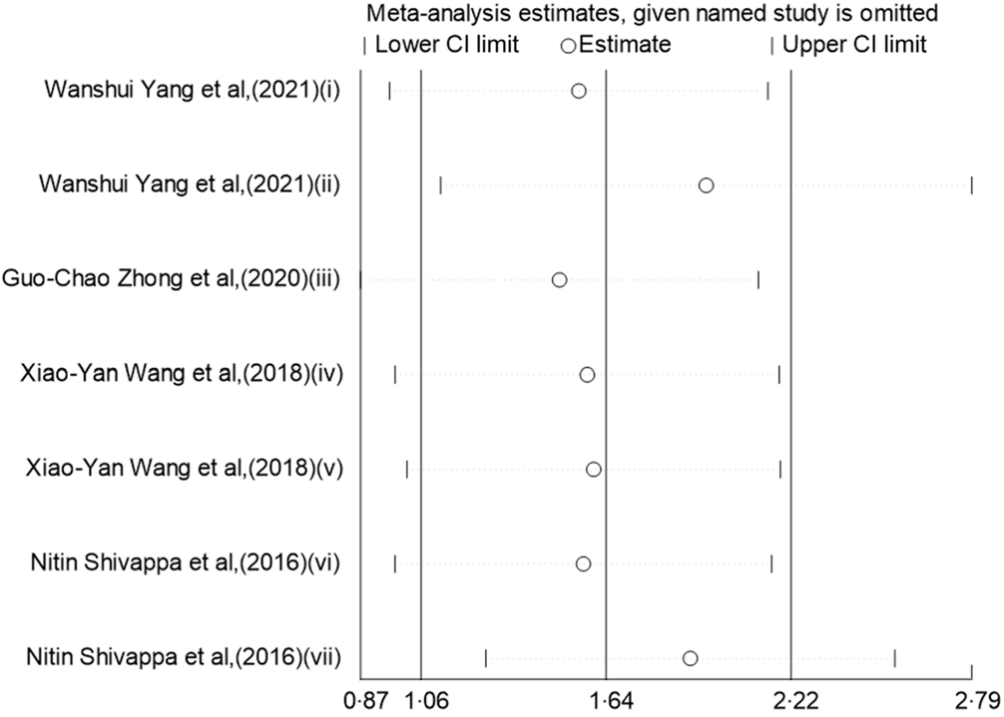
Sensitivity analysis was performed by removing each study in turn and recalculating the pooled relative risk (RR) estimates (categorical).

**Fig. 5:**
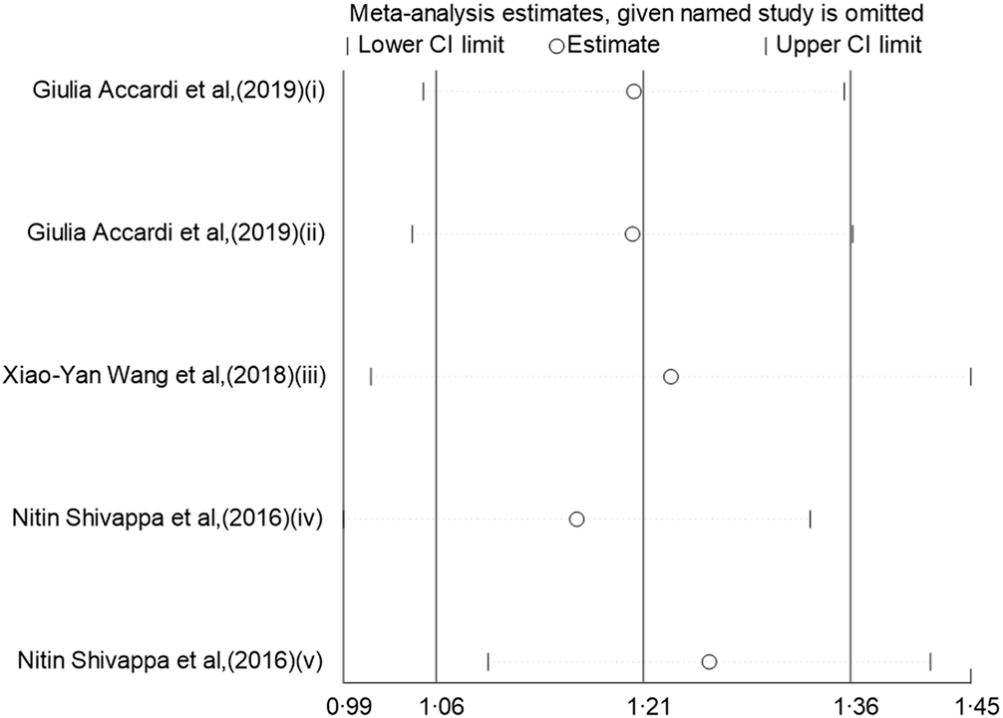
Sensitivity analysis was performed by removing each study in turn and recalculating the pooled relative risk (RR) estimates (Continuous).

In the sensitivity analysis of the categorical data studies (Fig. 4), excluding individual studies one by one did not significantly affect the results of the meta-analysis, indicating that this result is robust in terms of data sensitivity. The same result was obtained in the sensitivity analysis of the continuous data studies (Fig. 5).

### Subgroup analysis

**Fig. 6:**
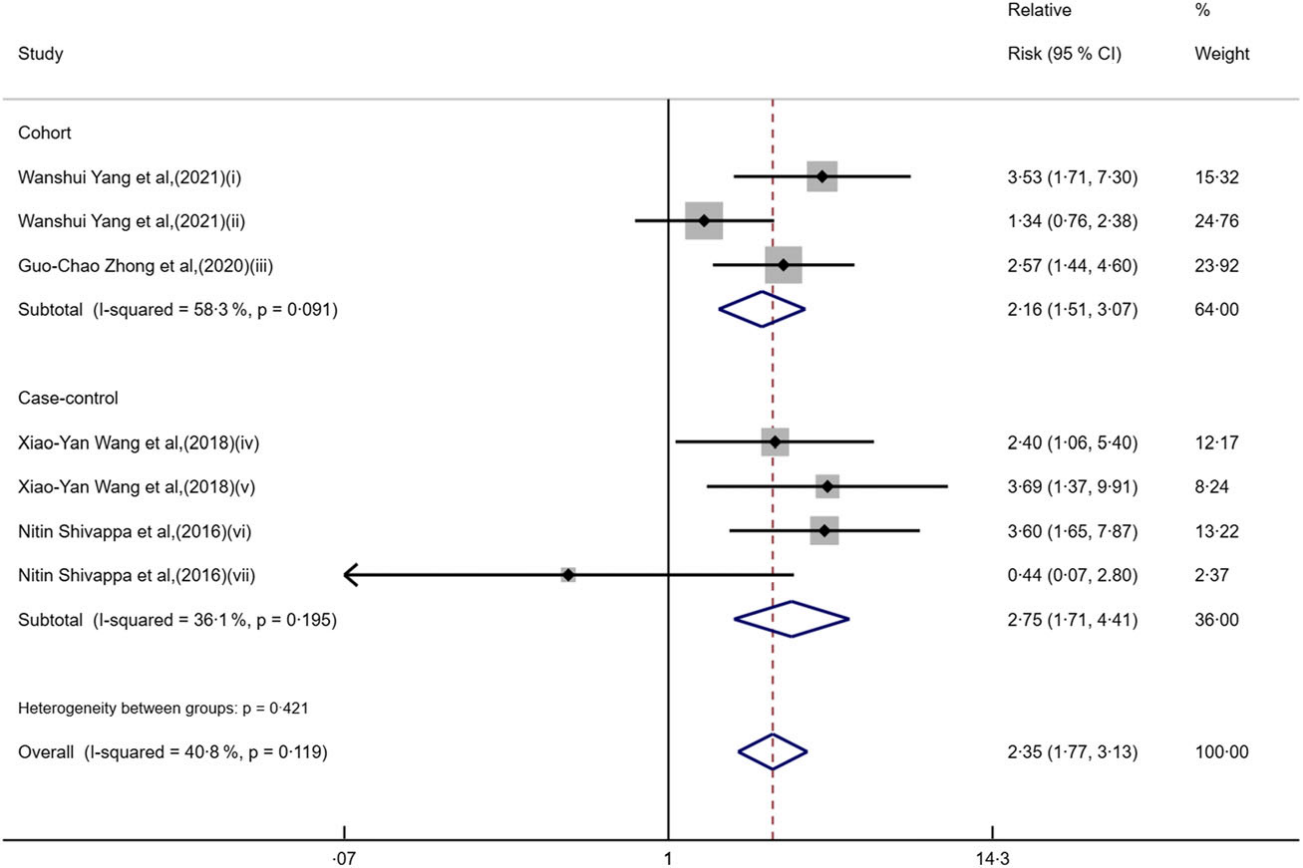
Forest plot for relative risk (RR) of subgroup analysis of study design.

In the categorical data study, we conducted subgroup analyses based on the study design, which suggested that cohort (I² = 58·3 %, *P* = 0·091) and case–control (I² = 36·1 %, *P* = 0·195) studies heterogeneity showed differences. In the cohort study subgroup, RR values indicated a 1·16-fold increased risk of liver cancer in the highest category of DII/EDIP compared to the lowest category of DII/EDIP (RR = 2·16; 95 % CI 1·51, 3·07; *P* = 0·000). Similarly, in the subgroup of case–control studies, RR also indicated that the highest category increased the risk of liver cancer compared with the lowest category (RR = 2·75; 95 % CI 1·71, 4·41; *P* = 0·000) (Fig. 6 and Table 2).

## Discussion

The present systematic review pooled five studies to assess the relationship between a pro-inflammatory diet and the risk of liver cancer. The outcomes revealed that a higher risk of liver cancer was associated with higher DII/EDIP scores. However, there was low heterogeneity among studies, which may be related to the diet index used. It may also be related to the study design; the populations of the different studies were different with different risks of liver cancer. More specifically, among the four original reviews that used DII calculations, two used data on thirty-one foods and nutrients for DII calculations, and the other two used thirty-two and thirty-five, respectively. These food parameters include ingredients and individual foods associated with inflammatory biomarkers (i.e. C-reactive protein, IL-4, IL-6, IL-10 and IL-1*β*, and TNF-*α*)^(17)^. For example, in different studies, total fat and alcohol have proved related to liver cancer. In a cohort study on China’s population, total fat was positively associated with liver cancer risk in men^(33)^ but did not show an association in women^(34)^. As well, alcohol had anti-inflammatory potential and a low DII score of –0·278/g, whereas excessive alcohol consumption can lead to increases in the risk of liver cancer^(35)^. The above studies suggest that individual nutrients or dietary components of the DII can have a direct impact on liver cancer, but it is important to note the different conditions. Our study shows that dietary inflammation in an individual is powerfully associated with liver cancer, reflecting the fact that dietary inflammation in an individual can have a stronger effect than a single nutrient or dietary component. This is also consistent with previous study^(36)^. Additionally, subgroup analysis indicated a significant positive association between high DII/EDIP and liver cancer in both cohort and case–control studies. However, additional subgroup analyses were not performed, and although a discussion of heterogeneity in BMI and physical activity is necessary for nutrition studies, there was a lack of consistency in the adjusted variables across the five included articles, so differences in risk factors were not explored further. The inclusion of the general population in our study also makes the risk of morbidity inconsistent across populations, such as those of different ages or sexes.

Previous review suggested a positive association between the dietary inflammatory pattern (*a priori* DII and data-driven dietary inflammatory patterns) and hepatocellular carcinoma (HCC) risk^(36)^. The results are consistent with our findings that DII/EDIP is associated with an increased risk of liver cancer. The differences are mainly in the population, alcohol exposure and outcome. In our study, the subjects are from the general population, and alcohol exposure was also taken into account. The outcome is primary liver cancer, which includes not only HCC but other types as well. Additionally, DII and EDIP were combined for analysis in this study. Consistently, this study confirmed a direct association between DII/EDIP and liver cancer and provided evidence for the selection of quantitative tools for dietary risk screening of liver cancer. This useful technique can help promote the dietary health management of ordinary people, by revealing the association between liver cancer and the risk of diet-induced inflammation.

DII can be used to validate the elevation of six inflammatory markers (i.e. IL-1*β*, IL-4, IL-6, IL-10, TNF-*α* and C-reactive protein). The IL-1*β* rs1143627 T allele enhances IL-1 production in the liver and induces injury to hepatocytes, which may result in the development of liver cancer^(37)^. This increased level of IL-4 activated the extracellular regulated protein kinases and serine/threonine protein kinase B signalling pathways to enhance the migration and invasion of liver cancer cells and further expansion of cancer stem-like cells^(38)^. The inflammatory cytokines TNF-*α* and IL-6 which cause hepatic inflammation and activation of the oncogenic transcription factor STAT3 drive liver cancer^(39,40)^. Additionally, IL-10 plays a crucial role in liver cancer, since the shift to Th1 pattern-like cytokines in the liver may result in more inflammation, necrosis of hepatocytes, and subsequent regeneration, which leads to mutagenesis and proto-oncogene activation in the host cells, leading to liver cancer^(41)^. High CRP level is a marker of an ongoing inflammatory process that favours tumour development. Necrotic cells release pro-inflammatory signals, which attract inflammatory cells from the surrounding tissue^(42)^. Therefore, elevated levels of these inflammatory markers (i.e. IL-1*β*, IL-4, IL-6, IL-10, TNF-*α* and C-reactive protein) increase the risk of liver cancer development. This is supported by our observation that dietary inflammatory potential significantly affects liver cancer risk.

This meta-analysis also has a few limitations. Above all, due to a lack of published data, there existed limited subgroup analyses; this is also caused by incomparable associations among the studies that employed different exposure or classification methods. Additionally, it is worth noting that our findings are based on cohort/case–control studies instead of randomised controlled trials, caused by the diversity of dietary exposures. Third, we did not do publication bias mapping because fewer than ten articles were included. However, our results provide evidence-based guidance to dietary health education, for potential prevention of liver cancer. In the future, relevant studies are required to further confirm the intrinsic causal relationship between the pro-inflammatory diet and liver cancer.

### Conclusions

This meta-analysis suggests that a higher dietary inflammatory potential (pro-inflammatory diet) can increase the risk of liver cancer, also consistent across study types and overall analyses. The contributions of a pro-inflammatory diet to liver cancer are a popular research area, but it requires further investigation to identify new and feasible intervention strategies. Even though the mechanism of inflammation in liver cancer has not been fully understood, this work sheds light on the prevention of liver cancer with the help of dietary targeting.
